# Country-level factors in a failing relationship with nature: Nature connectedness as a key metric for a sustainable future

**DOI:** 10.1007/s13280-022-01744-w

**Published:** 2022-05-31

**Authors:** Miles Richardson, Iain Hamlin, Lewis R. Elliott, Mathew P. White

**Affiliations:** 1 School of Psychology, University of Derby, Kedleston Road, Derby, UK; 2 European Centre for Environment and Human Health, University of Exeter Medical School, c/o Knowledge Spa, Royal Cornwall Hospital, Truro, Cornwall UK; 3 Cognitive Science HUB, University of Vienna, Kolingasse 14/16, 1090 Vienna, Austria

**Keywords:** Biodiversity, Indicators, Metrics, Nature connectedness, Sustainability

## Abstract

Climate change and biodiversity loss show that the human–nature relationship is failing. That relationship can be measured through the construct of nature connectedness which is a key factor in pro-environmental behaviours and mental well-being. Country-level indicators of extinction of nature experience, consumption and commerce, use and control of nature and negativistic factors were selected. An exploratory analysis of the relationship between these metrics and nature connectedness across adult samples from 14 European countries was conducted (*n* = 14,745 respondents). The analysis provides insight into how affluence, technology and consumption are associated with the human–nature relationship. These findings motivate a comparison of how nature connectedness and composite indicators of prosperity, progress, development, and sustainability relate to indicators of human and nature’s well-being. In comparison to composite indexes, it is proposed that nature connectedness is a critical indicator of human and nature’s well-being needed to inform the transition to a sustainable future.

## Introduction

Human-induced climate change and biodiversity loss show that the human–nature relationship is failing. That relationship between individual humans and nature can be described and measured through the psychological construct of nature connectedness, the closeness of an individual’s relationship with nature. Systematic reviews and meta-analyses have shown that nature connectedness is a key factor in the pro-environmental behaviours associated with addressing climate warming (Mackay and Schmitt 2019) and the pro-nature conservation actions that support biodiversity (Richardson et al. 2020a). Further, systematic reviews and meta-analyses have shown positive associations with mental well-being (Pritchard et al. 2020) with a causal link being evidenced (McEwan et al. 2019). Given the emerging importance of the construct to help address the global challenges of climate change, wildlife loss and mental well-being (Lambert et al. 2020; Dasgupta 2021), there is value in exploring the relationship between country-level metrics that may explain closer and more distant relationships with nature.

As a construct that can be measured using psychometric scales, nature connectedness provides a route to exploring the human-relationship quantitatively. Recently, a survey of 18 countries showed differing levels of nature connectedness (as measured using the INS; Schultz 2002), particularly in English speaking former British colonies which were revealed to have the lowest levels of nature connectedness (White et al. 2021). This raises the possibility that there are cultural and societal factors that may explain the failing relationship with nature that damages planetary well-being. Understanding country-level factors that are associated with individual levels of nature connectedness may help inform work to improve the human–nature relationship. Further, confirming an association between country-level factors and individual levels of nature connectedness would help confirm its value as an indicator of human and nature’s well-being. The initial analysis sets out to study how various country-level metrics are associated with nature connectedness before a comparison to composite indicators is made.

This exploratory approach is inspired by Wilkinson and Pickett (2010). Their book *The Spirit Level* was provocative, arguing that high levels of inequality in societies are harmful and that economic growth and income per person are not the most important predictors of thriving societies. Wilkinson and Pickett examined the correlation between income inequality and several indicators of inequality, including mental health, obesity, teenage births, crime and education performance. The consistent relationship to income inequality was used to argue that income inequality was accountable for societal ills. Although there are analytical limitations, which will be covered later, the approach, especially with balanced interpretations, enables debate and some insight that can motivate and inform further, more robust studies.

A key first step to this approach is the selection of exploratory country-level indicators that might be related to lower levels of individual nature connectedness, the factor of interest. The framework for indicator selection used was Kellert’s (1993) typology of the values of biophilia. Although biophilia is often considered as a theory regarding an innate love of nature, it also reflects the innate human need for nature for survival. Thus, when considered at scale, Kellert’s values include both positive and negative relationships with the natural world. Using these nine values as a framework, the pathways to nature connectedness (Lumber et al. 2017) identified five values associated with higher levels of nature connectedness. Richardson et al. (2020b) proposed that three of the remaining four types of relationships with nature, those unrelated to nature connectedness, had a clear negative impact on the natural environment and are likely to be related to a more distant relationship with nature, especially when they are dominant. These types of relationship were utilitarian (use of natural resources), dominionistic (control of nature) and negativistic (fear of nature).

These types of relationship are common, with use of natural resources and fear, at a certain level, essential for human survival. However, through agricultural, scientific and industrial revolutions, the control and use of nature has accelerated (Wiedmann et al. 2020). The dualistic foundations of the scientific revolution strengthened separate concepts of human and nature, and the post-Enlightenment worldview reconceptualised nature into a catalogue of resources (Hamilton 2002). Since the Industrial Revolution, there has been a perceived right of exploitation of natural resources (Weber 1968) and global growth in affluence relies upon continuous increases in resource use. Further, such growth is seen as an indicator of progress that delivers greater prosperity and happiness (Eckersley 2000). Therefore, in addition to indicators of utility and dominion, indicators of the purpose of such relationships, consumption and commerce were also identified and included in the present analysis. Finally, as introduced below, there is a strong argument to include indicators of ‘extinction of experience’. These four groups of nature relationships and factors will differ between cultures and countries and persist through to the present day.

In sum, the discussion above proposes four indicator groups: extinction of nature experience, consumption and commerce, use and control of nature and negativistic factors. These inform an exploratory analysis of the relationship between these factors and nature connectedness. The specific indicators for each group measured across 14 European countries are now introduced and justified (for summary see Table 1). The specific metric for each specific indicator is stated in the method section.

**Table 1:** Indicator groupings and country-level metrics

Indicator group	Metric
Extinction of nature experience	Urban population percentage Population aged 65+ percentage
Consumption and commerce	Average personal income Energy use Smartphone ownership
Utility and dominion	Cultivated land percentage Biodiversity (National Biodiversity Index) Material footprint
Negativistic	Natural disaster vulnerability Average annual rainfall

### Extinction of nature experience

Extinction of experience of nature (e.g. Pyle 2003; Soga and Gaston 2016) is often related to levels of nature connectedness and is associated with urbanisation. Extinction of experience is discussed in terms of two key factors, the loss of orientation towards engaging with nature and loss of opportunity to experience nature. Increased urbanisation drives the loss of opportunity to experience nature directly by reducing the quantity, quality and diversity of natural spaces (e.g. Lekies and Brensinger 2017; although see Oh et al. 2020; Novotný et al. 2021). Research shows that direct experience, simple engagement and noticing nature are key to developing nature connectedness (McEwan et al. 2019; Richardson et al. 2022). As urban populations live in built environments which typically have less green space, nature and biodiversity (Miller 2005), there is less nature to notice and engage with and therefore we would expect to see lower levels of nature connectedness in countries with more people living in urban environments (Soga and Gaston 2016). Therefore, a country-level metric for urban population is included as the first indicator for extinction of experience.

Further, loss of wildlife creates a shifting baseline syndrome where each generation accepts current levels of nature as the norm (Papworth et al 2009; Soga and Gaston 2018). Such loss of wildlife and therefore opportunity to engage with nature permeates culture and becomes a social norm (Nyborg et al. 2016). Therefore, given the 60% decline in wildlife since 1970 (WWF 2018), populations with a higher proportion of older adults who had the opportunity to engage with more wildlife and develop a closer relationship with nature could still influence social norms such that adult levels of nature connectedness more generally are higher. Research shows that levels of nature connectedness are lower in young adulthood (Richardson et al. 2019), but intergenerational transmission of nature connectedness does occur (D'amore 2016). Therefore, a metric for proportion of adults aged 65 years and over was included as the second indicator for extinction of experience. The two metrics will be used to explore the potential for an association with nature connectedness.

### Consumption and commerce

As introduced earlier, White et al. (2021) found that a cluster of English speaking, former British colonies had relatively low levels of nature connectedness compared to other societies such as Southern European, Eastern European or Nordic countries. There is a notable difference in economic growth across these regions. Western European and Western offshoots have seen large-scale economic growth since 1820, with personal income increasing 13-fold and 17-fold, respectively, compared to sixfold in Eastern Europe and tenfold in Southern Europe (Maddison 1995). Levels of affluence are drivers of consumption and unstainable trends (Wiedmann et al. 2020). Therefore, these differentials motivate the inclusion of indicators to explore potential associations around consumption and commerce to nature connectedness.

Personal income is an indicator of consumption (Wiedmann et al. 2020). Income reflects both a country’s economic position and the individual’s ability to spend on consumer goods. The consumerism facilitated by income also reaches beyond purchasing goods to personal enhancement, with the result that marketing can attempt to make people both dissatisfied with what they have and who they are (Eckersley 2000); with the targeted purchase supposedly leading to happiness. However, a side effect of promoting consumer and technological route to satisfaction is that other sources of satisfaction, such as a close relationship with nature, can become overlooked. This is especially the case in the wider cultural context of the dualistic Western mindset that justifies and enables the exploitation and separation of people from nature (Hamilton 2002). Yet, nature connectedness has been found to explain levels of eudaemonic well-being nearly four times more than socio-economic status (Martin et al. 2020). A country-level metric of personal income is included as the first indicator for consumption and commerce.

The economic development that drives income and consumption depends upon energy use (Ahmed and Shimada 2019), although energy use is falling in industrialised nations and has become decoupled from GDP, with energy use falling in industrialised countries in recent decades (Mielnik and Goldemberg 2000). However, it is still a key factor in assessing sustainability given the reliance on fossil fuels (Jorgenson et al. 2014). Like income, energy use per capita was included as a broad exploratory indicator that reflects consumption, and economic development. A country-level metric of energy use is included as the second indicator for consumption and commerce.

Finally, we use smartphone penetration as an exploratory indicator for commerce and consumption. As well as being linked to improving well-being through purchase of goods (Hamilton 2002), the reduction in the meaning of nature in people’s lives has been linked to increases in consumer technologies (Kesebir and Kesebir 2017). Kesebir and Kesebir (2017) identified a cultural shift away from nature in an analysis of twentieth century works of popular culture. This analysis found that rather than rates of increasing urbanization, the decline of nature words was best explained by the arrival of new technologies. The rapid rise and increasingly widespread penetration of smartphones may have seen the arrival of another wave disconnection from nature, especially through uses such as social media, which reflects and rapidly shapes culture itself (Kesebir and Kesebir 2017). Further, technology is often cited as potential cause of a weaker human–nature relationship (Vanderburg 2000) and a human tendency to focus on activities involving electronic media has been identified (Pergams and Zaradic 2006). Finally, Richardson et al. (2018) found that people with higher smartphone use had a significantly lower nature connectedness score.

### Utility and dominion

Utilitarian values can provide the foundation for conflict with wildlife (Teel and Manfredo 2010), and utilitarian and dominionistic human–nature relationships are key to the excessive consumption that is a major cause of global environmental degradation (World Commission on Environment and Development 1987). In industrial and modern society, dominion over, and utilitarian use of nature ultimately produce prosperity and economic growth which are seen as signs of progress. This ever-increasing control and exploitation of nature and industrialisation is accompanied with the promise of prosperity and happiness (Eckersley 2000).

The potential influence of dominionistic control of nature was explored here by the selection of the amount of cultivated land as the first indicator of the utility and dominion group. While research into the psychological construct of nature connectedness explores individual relationships, as the psychometric tools are based on individual measurement, there is a body of literature on human–nature connectedness at the landscape scale, although this too can rely on individual level analysis through interviews, for example (Balázsi et al. 2019). Landscape change has been found to have a strong, and often negative, influence on human–nature connectedness (Riechers et al. 2020), with land use intensity impacting the local population materially, experientially, and emotionally (Balázsi et al. 2019), which is key for psychological nature connectedness.

Given the known relationship between land cultivation and biodiversity loss while also recognising the role played by various other geographical and ecological factors, biodiversity was included as the second indicator of the utility and dominion group. Where utilitarian and dominionistic relationships with nature dominate (e.g. greater levels of cultivated land), there may be less natural habitat and less wildlife, be that flora or fauna (Pereira et al. 2012). There is little research that directly examines nature connectedness and biodiversity, yet there is related research that suggests an association (Hoyle et al. 2019). When prompted, people are good at estimating levels of certain types of biodiversity across various habitats (Cameron et al 2020), and a relationship to nature connectedness is likely owing to improved levels of nature connection being built through noticing nature (McEwan et al. 2019).

A final indicator of the utility and dominion group and used to explore how utility and dominion relates to nature connectedness was the broad area of mineral production. This is quite a literal and direct indicator of utilitarian use of natural resources. To represent the use of natural resources, the material footprint for each country was used (Wiedmann et al. 2015). This is a consumption-based indicator of resource use, including raw material extraction and demand. When considering mineral extraction, there is the complexity of political pacification, social manipulation and conflict surrounding land deals that remains under acknowledged (Dunlap 2020). Although a major issue is in areas outside the current sample (e.g. countries within Africa and Latin America; Hogenboom 2012), such issues are present in Europe. Dunlap notes how the world’s largest opencast lignite mine in Germany is expanding into a highly biodiverse forest on land ‘grabbed’ by the state. Dunlap also notes the normalization of resource extraction for economic growth and acceptance of the situation by researchers. Care is needed so that the social manipulation used to support activities such as mineral extraction by global conglomerates is not overlooked. Therefore, the present analysis may reveal relationships that are outside the individuals’ control, but nonetheless it is an important issue to explore as the impacts of such practices could be harming populations with a close relationship with nature, damaging that relationship, or creating a barrier to such relationships developing. These could inform the governance of extraction for more inclusive development (Bebbington 2013).

### Negativistic

Finally, country-level indicators were also required for the negativistic grouping. These were included to tap into Kellert’s negativistic relationship type as there is evidence of relationship between higher levels of fear of nature and lower levels of nature connectedness (Zsido et al. 2022). For a country-level indicator, the risk of natural disasters was included as an exploratory indicator of potential fear of nature. Experiences of natural disasters have been found to alter the human–nature relationship (Brown 2017). Therefore, countries at higher risk of natural disasters may have lower levels of nature connectedness. At a less exceptional level, poor weather could also impact the human–nature relationship through reducing the enjoyment of nature (Elliott et al. 2019). Therefore, to control for the potential impact of the weather on levels of nature connectedness, country-level rainfall was also included as the indicator for the negativistic indicator grouping.

### Nature connectedness as an index

The strong evidence of the benefits to mental well-being from increased nature connectedness has seen nature connectedness identified as a basic psychological need (Baxter and Pelletier 2019; Hurly and Walker 2019). This is reflected by the inclusion of nature connectedness as a wellbeing indicator in the Gallup World Poll (GWP), an internationally respected tool for global decision-makers interested in topics such as health, wellbeing and sustainability (Lambert et al. 2020; Gallup n.d.). When the causal relationship to pro-environmental behaviours is added in addition, nature connectedness becomes an even more attractive target for improvement, but also monitoring as a key sustainability and well-being metric, unique in that it sits at the interface of people and nature’s well-being. Measured by a single item question in the current analysis, the construct of nature connectedness reflects the human–nature relationship within fragmented thinking and can unite human and nature’s well-being.

In the following analysis, the associations between the ten country-level indicators and nature connectedness will be followed by a comparison of how nature connectedness and country-level composite indicators of prosperity, social progress, human development and sustainability relate to indicators of human and nature’s well-being. This comparison and macro view of country-level factors related to individual relationships with nature is intended to stimulate debate and prompt new approaches to improving and reporting the human–nature relationship for a sustainable future.

## Materials and methods

### Single country-level metrics

Country-level measures for each of the ten indicators listed in Table 1 were identified where possible and the relationship of those indicators to each country’s level of nature connectedness was calculated. Nature connectedness data were gathered in the BlueHealth survey (Elliott and White 2020) and presented by White et al. (2021). The BlueHealth survey included the Inclusion of Nature in Self (INS; Schultz 2002) scale which asks respondent to rate their interconnectedness with nature by selecting one of seven pairs of circles which overlap to a varying degree. There are several tools that measure the concepts around nature connectedness, and Tam (2013) found strong convergence between them and that they, including the INS, measured a higher order common construct. The INS is often used in studies measuring nature connectedness and referred to as a nature connection measure (e.g. Kleespies et al. 2021). Further, results from the INS and other measures have been combined in meta-analyses of nature connectedness (e.g. Pritchard et al. 2020), suggesting that our operationalisation here with the INS is in keeping with the broader field.

The survey was conducted online between June 2017 and April 2018, to include sampling across all four seasons, and across 18 countries. Three of the countries surveyed by White et al. (2021) had a regional sample (California, Hong Kong and Queensland) so a comparison to country-level indicators is not ideal. As this left Canada as the only non-European country, it was decided to focus on the remaining 14 European countries (United Kingdom, Republic of Ireland, Netherlands, Finland, Germany, Sweden, Estonia, Greece, France, Spain, Bulgaria, Portugal, Italy and the Czech Republic) where stratified broadly demographically representative samples of ≈ 1000 adult respondents were collected in each country. This represents a more homogeneous group, which in addition to providing a sound rationale would suggest any emerging relationships between the indicators and nature connectedness will be more meaningful. Full details on the sample and survey items can be found in Elliott and White (2020).

The country-level metrics for each of the ten indicators listed in Table 1 were as follows:

- Biodiversity: Measured using National Biodiversity Index. This index is based on estimates of country richness and endemism in four terrestrial vertebrate classes and vascular plants; vertebrates and plants are ranked equally (Convention on Biological Diversity 2022).
- Population aged 65+: Percentage of the population aged 65+  years old (World Bank 2019).
- Cultivated land: Percentage of land under cultivation, arable plus permanent crops, not pasture (CIA n.d.).
- Natural disasters: Vulnerability to natural disaster measured in the World Risk Index. Calculated by the United Nations University Institute for Environment and Human Security (UNU-EHS 2016).
- Material Footprint: Index that includes construction materials, metal ores and biomass (Wiedmann et al. 2015).
- Energy Use: kWh per capita from IEA Statistics (World Bank 2022a).
- Urban population: Percentage of total population living in an urban area (UN 2018).
- Rainfall: Average precipitation in depth (mm per year) from Food and Agriculture Organisation (World Bank 2022b).
- Income: Mean income per capita from Global Database of Shared Prosperity (World Bank 2022c).
- Smartphones: percentage of population actively using a smartphone (Newzoo 2019).

### Composite indices

The value of using nature connectedness as an indicator was explored by comparing its association to key measures of human and natural environmental wellbeing in comparison to indicative composite indices of prosperity, human development, sustainability and social progress across the 14 countries.

As above, nature connectedness was measured using data from White et al. (2021) using the INS. Well-being was also measured using data from White et al. (2021) who, following previous studies (Mitchell et al. 2015; Garrett et al. 2019), used the World Health Organisation’s 5-item index of positive well-being. As above, biodiversity data were from the National Biodiversity Index. CO2 emissions by country were from the Emissions Database for Global Atmospheric Research (EDGAR) and include all human activities leading to climate relevant emissions, except biomass/biofuel combustion (European Commission et al. 2019). Four indicative composite indices of prosperity, human development, sustainability and social progress were selected:

- The UN Human Development Index (HDI) assesses the development of a country based on the people and their capabilities rather than economic growth. The HDI incorporates life expectancy at birth, years of schooling and gross national income per capita into a single index (UNDP 2022).
- The Legatum Prosperity Index (LPI) is more complex, with 66 different elements, measured by nearly 300 discrete country-level indicators of key characteristics such as inclusive societies, open economies and empowered people (Legatum Institute 2021).
- The Social Progress Index (SPI) measures 50 social and environmental indicators. These include measurable outcomes such as shelter, nutrition, rights and education (SPI 2021).
- The Sustainable Development Report (SDR) ranks UN member states through a score that measures a country's overall progress towards achieving the 17 Sustainable Development Goals (SDG). The score can be interpreted as a percentage of SDG achievement such that a score of 100 indicates that all SDGs have been achieved (Sachs et al. 2021).

## Results

### Single country-level metrics

**Fig. 1:**
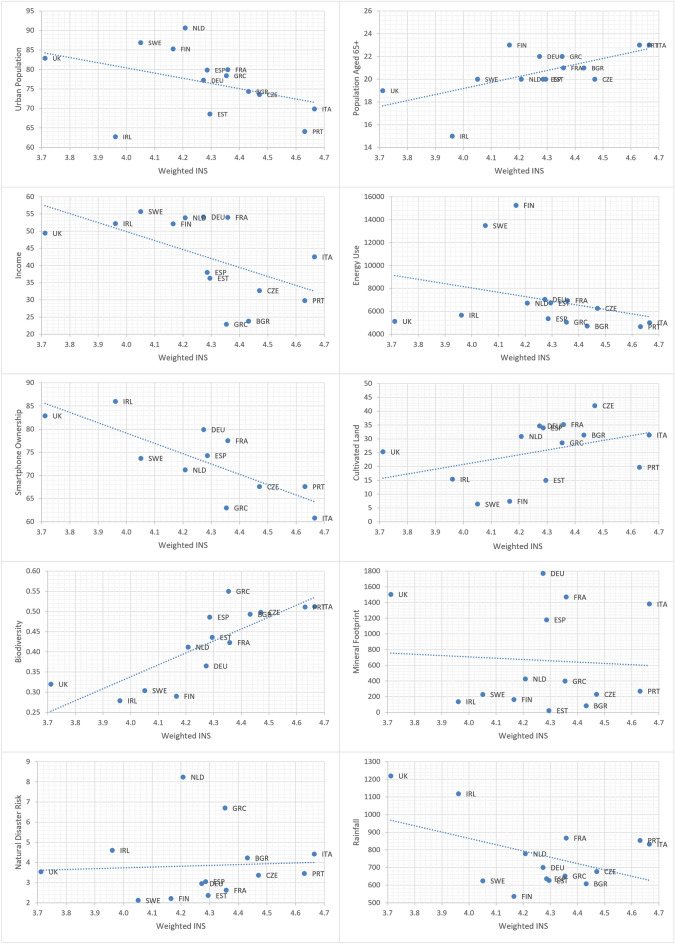
Charts showing association to nature connectedness for each country-level metric (*UK* United Kingdom, *IRL* Republic of Ireland, *NLD* Netherlands, *FIN* Finland, *DEU* Germany, *SWE* Sweden, *EST* Estonia, *GRC* Greece, *FRA* France, *ESP* Spain, *BGR*  Bulgaria, *PRT* Portugal, *ITA* Italy, *CZE* Czech Republic)

Ten correlations were undertaken to establish the strength of association between nature connectedness and each of the country-level metrics justified above and summarised in Table 1. The results of the correlations are presented in Table 2 and as charts in Fig. 1. This approach followed and was inspired by the approach of Wilkinson and Pickett (2010), who focussed on plotting countries in terms of income inequality on their horizontal axes and an array of country-level aggregate outcomes on their vertical axes (e.g. life expectancy, health, trust).

**Table 2:** Summary of the correlations between nature connectedness and each country-level metric

Metric	Correlation to nature connectedness	Metric	Correlation to nature connectedness
Biodiversity	0.806	Energy use	− 0.295
Proportion aged 65+	0.640	Urban population	− 0.402
Cultivated land	0.400	Rainfall	− 0.457
Natural disasters	0.059	Income	− 0.555
Material footprint	− 0.065	Smartphone penetration	− 0.784

In the current context, income inequality was replaced by nature connectedness on the horizontal-axis so that societies with higher levels of nature connectedness are towards the right of each chart. The different indicators are placed on the y-axis. Each chart shows a scatter of countries which reveals how different countries compare to each other. A ‘best fit’ trendline is included. Where the line slopes upwards from left to right, it shows country-level indicators that are more common in more nature connected societies. Where the line slopes downwards from left to right, it shows country-level indicators that are more common in less nature connected societies. A wider scatter of points (and more horizontal trend line) on the chart shows that there is not as strong a relationship between the human-nature indicator and nature connectedness.

### Composite indices

To explore the value of using nature connectedness as an indicator of the health of the human–nature relationship, it was compared to composite indices of prosperity, human development, sustainability and social progress. Correlations between the composite indices scores across the 14 countries to wellbeing, biodiversity and carbon emissions and nature connectedness are presented in Table 3. It can be seen that the composite indices all had a negative relationship to wellbeing, biodiversity and nature connectedness, whereas nature connectedness had a positive relationship to wellbeing and biodiversity. The relationship of all measures to carbon emissions was weak.

**Table 3:** Summary of the correlations between composite indices for each country and outcomes of human and nature’s wellbeing

	Nature connectedness	Carbon emissions	Well-being	Biodiversity
Legatum Prosperity Index	− 0.61	0.13	− 0.52	− 0.83
Human Development Index	− 0.65	0.06	− 0.51	− 0.76
Social Progress Index	− 0.44	0.01	− 0.33	− 0.67
SDR Rankings	− 0.38	0.20	− 0.42	− 0.72
Nature Connectedness	1.00	0.02	0.64	0.81

## Discussion

Country-level indicators were selected that could affect the human–nature relationship. The results show that three broad indicator groupings of extinction of nature experience, consumption and commerce, and utility and dominion included metrics that had a strong or moderately strong relationship to nature connectedness at the country aggregate level. This suggests that the overall approach to the justification of the indicators was sound, and that extinction of experience, consumption and commerce and utility and dominion are country-level factors that are linked to the individual human–nature relationship.

Across all the metrics, biodiversity and smartphone penetration had the strongest relationships to nature connectedness, with income and proportion of older adults providing the next strongest relationships (Table 2), with all four showing correlations associated with strong effects (Richard et al. 2003). Biodiversity emerged as having the strongest relationship with nature connectedness. It is helpful that this is likely to be a reciprocal relationship, with higher level of biodiversity being linked to a close relationship with nature and in return a close relationship with nature a key factor in pro-nature conservation behaviours. Capturing this key relationship supports the use of nature connectedness as an important indicator used to plot and monitor moves to a more sustainable future.

### Extinction of nature experience

The indicators grouped to indicate extinction of nature experience (as shown in Table 1) showed a moderate relationship to nature connectedness (as shown in Table 2). A greater level of urban population is moderately associated with lower nature connectedness, possibly as some areas with high urbanisation have relatively few ‘green’ urban areas (Cameron et al 2020). The data support that the concern that people living urban lives are surrounded by fewer natural habitats which reduces the variety of wildlife and opportunities to experience nature (Pyle 2003; Miller 2005; Soga and Gaston 2016). From a nature connectedness perspective, there is less opportunity for the sensory contact, noticing of nature’s beauty, emotional and meaningful engagement and opportunity to care for nature that form the pathways to nature connectedness (Lumber et al. 2017). Specifically, the noticing of nature known to build nature connectedness (Richardson et al. 2022). This also reduces the orientation to engage with nature, impacting emotional affinity with nature (Soga and Gaston 2016) which is then reflected in cultural feedback, through the decline of references to nature in cultural products, for example (Kesebir and Kesebir 2017). With fewer natural habitats, reinforced by cultural feedback and attention directed to other consumer experiences, a more distant relationship with nature becomes the social norm.

The proportion of older adults in the population had a moderately strong relationship to higher levels of nature connectedness. The data support the notion that the shifting baseline syndrome suggests older generations had a greater experience of nature and therefore opportunity to engage with nature and build a closer connection to it. The result suggests that a society with more older adults is a more nature connected society, showing the potential value of intergenerational programmes to increase nature connectedness (D'amore 2016).

### Consumption and commerce

Two metrics for consumption and commerce had moderately strong negative relationship with nature connectedness. Higher levels of income were moderately associated with lower levels of nature connectedness. The data support the proposition that personal income is an indicator of consumption of resources that can separate people from nature (Hamilton 2002; Wiedmann et al. 2020). The pursuit of happiness through prosperity is well established in consumerist societies (Eckersley 2000), although diminishing returns have been suggested (Diener and Diener 1995) and refuted (Stevenson and Wolfers 2008). Either way, the relationship between higher income and lower levels of nature connectedness is interesting, especially when socio-economic status has been found to explain levels of eudaemonic well-being nearly four times lower than nature connectedness (Martin et al. 2020). The paradox being that pursuit of well-being through economic growth is likely to have a negative relationship to nature connectedness which explains greater levels of well-being. As well as the role of income in a more distant relationship with nature, this debate promotes the use of nature connectedness as a metric for both well-being and a sustainable future.

There was a strong relationship between higher levels of smartphone ownership and lower levels of nature connectedness. The cross-country analysis fits findings at the individual unit of analysis where people with higher smartphone use had lower nature connectedness scores (Richardson et al. 2018). As well as an indicator of consumerism, the data highlight the need to consider the role of technology in the human–nature relationship, both in terms of the human tendency to focus on electronic media and cultural shifts away from nature (Pergams and Zaradic 2006; Kesebir and Kesebir 2017). Especially as technological development will continue, such as creating immersive environments for well-being and profit (Huang 2021). Technology has reduced the impact of disease and hunger and delivered wonderful progress, but technology has increased the threat to the ecology that sustains human life (Schmidt and Marratto 2008).

The final consumption and commerce indicator was energy use. There was a weak relationship, with higher levels of power consumption associated with lower levels of nature connectedness. This relationship could be weaker as a colder climate necessitates higher consumption (i.e. Finland, Sweden). Further, the factors involved in the level of energy use are complex, with individual control (Chen and Sintov 2016), and counter intuitive relationships, such as urbanisation being associated with lower levels of energy use (Sadorsky 2014). The wider scatter of points on the chart reflects this range of important influences. Finally, the link between energy use and carbon emissions loosens as countries adopt renewable energy sources.

### Utility and dominion

Levels of biodiversity are an ultimate reflection of the excessive consumption that causes the degradation of the natural environment, and a strong positive relationship was found between biodiversity and nature connectedness. The direction of causality and whether it is the loss of wildlife that damages the relationship, or the weakening relationship that leads to the loss of wildlife, is somewhat a moot point as neither is beneficial. Further, it is likely that there is a reciprocal relationship between biodiversity and nature connectedness given the link between nature connection and ‘habitat creating’ behaviours (Richardson et al. 2020a) and high biodiversity providing more nature to notice thus building nature connectedness (McEwan et al. 2019) and further pro-nature conservation behaviours (Richardson and Hamlin 2021).

It should be noted that the National Biodiversity Index is based on estimates of richness in four terrestrial vertebrate classes and vascular plants. The index reflects the whole country when people tend to live mainly in urbanised areas where biodiversity tends to be lower (Cameron et al. 2020), yet there are more people to engage with the nature that may be present. The likelihood that biodiversity may be higher in population centres within countries with higher levels nationally is beyond the scope of the paper, but the strong positive relationship between higher levels of biodiversity and nature connectedness suggests this may be the case.

The proportion of cultivated land was included as an indicator of use and control of nature within each country. In our analysis higher levels of cultivated land are moderately positively related to higher levels of nature connectedness. This unexpected relationship has several possible explanations. First, there are many ways of cultivating land, the measure used may not represent industrial farming, or any changes may have occurred slowly resulting in less negative impact (Riechers et al. 2020). Small scale, more organic cultivation could use a similar, or indeed larger amount of land, as more is required for similar returns. Such organic cultivation might be a positive in the human-–nature relationship with greater levels of community farming associated with higher levels of nature connectedness (Pérez-Ramírez et al. 2021). Further, the analysis measured arable land, a focus on pasture reveals a negative relationship. Finally, even large fields with tidy hedgerows can look like a “natural” landscape to some people and may be viewed positively as the landscape of their ‘home’ (Balázsi et al. 2019).

Collective farming develops a wider sense of place and has the potential to enhance human-nature connectedness through introducing nature into people’s daily lives (Pérez-Ramírez et al. 2021). It is interesting to note the farming and cultivation practices of the countries in the top-right quadrant of the scatterplot for cultivated land and nature connectedness. For example, Bulgaria has a history of state-run collective farming and in France cooperative agriculture accounts for a significant proportion of the national food industry's production. Finally, in Spain, the regional Madrid government introduced the public initiative of participatory agricultural laboratories in order to use agrarian activities to restore the relations between rural, peri-urban, and urban areas and the natural environment. This initiative explained higher levels of nature connectedness (Pérez-Ramírez et al. 2021). Interpretation is highly speculative at this point but the links between land use and nature connectedness shows the need for further investigation. Nature connectedness research has tended to focus on local interventions and there is clear potential for those working in domains such as psychology, sustainability, and socio-ecological systems to work more closely together, combining methods and metrics.

The final utility and dominion indicator is the material footprint of each country. There was no real relationship to nature connectedness. Although a direct and literal indicator of utilitarian resource use, measures of natural resource are not straightforward. If imported, domestic mineral use may have less direct impact on the population. However, focussing on local extraction that may impact the population is dependent on the availability of local resources. Indicators of natural resource use that impact on the individual level of nature connectedness require further exploration.

### Negativistic

Natural disasters and rainfall were included as indicators of potential negativistic relationships with nature. A fear of nature could arise through more regular extreme weather events and rainfall can impact on the opportunity and orientation to experience nature. We were unable to identify a suitably diverse metric of risks to well-being from wildlife across Europe (e.g. number of poisonous species), but the risk of natural disasters indicator had no relationship with nature connectedness. However, there was a moderate relationship between higher levels of rainfall and lower levels of nature connectedness, reflecting perhaps reduced opportunities to engage positively with nature, or engagement that is compromised in comparison to drier climates (Hartig et al. 2007). Although a moderate relationship was found, a key aspect is that climate factors outside human influence relate less strongly to nature connectedness than income, smartphone penetration and biodiversity.

### Limitations

Rather than a formal research paper, the straightforward exploratory analysis across multiple countries is intended to provide a unique perspective and prompt discussion. However, limitations of the approach should be noted. Correlation does not show causation; however, the use of several indicators around a common theme can bring consistency that can support causal inference (Pickett and Wilkinson 2015). Several of the potential causal indicators listed also come before the effect (nature connectedness). National indicators such as urbanisation, land use, biodiversity, energy use and income have a long gestation that can affect individual perceptions of nature connectedness. Causation can also be suggested when there is other evidence of causal effects, for example, increased nature experience increasing nature connectedness. However, it may still be the case that a long-standing culture of nature connectedness in a country brings about conditions which give rise to higher biodiversity, less urbanisation and lower income. Further, the relationships may not necessarily be linear or constant and the correlations may be caused by a third factor exerting an effect indirectly (Liebig 2012). Hence, the analysis is exploratory and interesting relationships will need to be followed by more sophisticated analysis, potentially of a wider group of countries. Further still, care should be taken when inferences about individuals are deduced from group data. However, research has been included to show that there is evidence at the individual level for the key relationships, for example biodiversity (Hoyle et al. 2019) and smartphone use (Richardson et al 2019). Finally, regarding limitations, from a global perspective, although there is a clear variation between countries in the current analysis, all are high-income countries apart from Bulgaria. However, the presence of moderate to strong relationships in this more homogenous group and of country-level indicators to individual levels of nature connectedness has shown value in the approach. Identifying a relationship between country-level indicators and individual levels of nature connectedness in either direction is important.

### Nature connectedness as an index

Building on its inclusion in the Gallup World Poll (Lambert et al. 2020) and the causal relationship to pro-environmental behaviours, the analysis above supports the use of nature connectedness as an indicator of the health of the human–nature relationship. Given the results, and simplicity of the nature connectedness metric, it is interesting to compare it to composite indices of prosperity, human development, sustainability and social progress.

Interestingly, whereas nature connectedness is positively associated with well-being, the four composite metrics all have a negative relationship to well-being and, more notably for the SDR ranking, biodiversity. Further, the selected composite indexes all have a negative relationship to nature connectedness, even the SDR ranking. Even though higher scores on these composite indexes are intended to reflect positive outcomes related to meeting basic human needs, human development, prosperity, and sustainability, they fail to capture the bond between people and nature that is emerging as essential for a healthy and sustainable life. The composite indexes are essentially anthropocentric with higher scores strongly related to lower levels of biodiversity. We propose nature connectedness as a critical, yet simple indicator of human and nature’s well-being to inform the transition to a sustainable future.

## Conclusion

The human–nature relationship is failing, leading to human-induced climate change and biodiversity loss. The simple yet powerful country-based analysis above strongly suggests that this failing relationship is related to affluent, technological consumer-based economies that consume natural resources and reduce biodiversity, which feeds back to further weaken the relationship through the shifting baseline syndrome. Further, the analysis suggests that the human–nature relationship, captured by nature connectedness, reflects both human and nature’s well-being better than some composite indexes.

The strong link between nature connectedness and well-being and that nature can bring more meaning to life than prosperity should be promoted. There is also a need to be more aware of the impact of technology, especially emerging immersive environments which could accelerate the extinction of ‘real’ experience, related to biodiversity loss and urbanisation.

Nature connectedness has often been considered at the level of the individual, with programmes and interventions to bring people closer to nature. However, the analysis above also shows the need for macro perspectives. The way land is used, how people engage with that land and how that land conserves nature and brings biodiversity to the population matters for the human–nature relationship. The nature of society, the nature of its economy, urbanisation and intergenerational activity are also related to the human–nature relationship. This unique country-level analysis adds power and direction to the need for a new relationship with nature for a sustainable future.
